# Divergence in wine characteristics produced by wild and domesticated strains of *Saccharomyces cerevisiae*

**DOI:** 10.1111/j.1567-1364.2011.00746.x

**Published:** 2011-09-02

**Authors:** Katie E Hyma, Sofie M Saerens, Kevin J Verstrepen, Justin C Fay

**Affiliations:** 1 Evolution, Ecology and Population Biology Program, Washington UniversitySt. Louis, MO, USA; 2 Department of Genetics, Center for Genome Sciences and Systems Biology, Washington UniversitySt. Louis, MO, USA; 3 Laboratory for Systems Biology, VIB, Bio-IncubatorLeuven, Belgium; 4 Laboratory for Genetics and Genomics, Centre of Microbial and Plant Genetics K.U. LeuvenLeuven, Belgium

**Keywords:** wine, aroma, flavor, fermentation, domestication, yeast

## Abstract

The budding yeast *Saccharomyces cerevisiae* is the primary species used by wine makers to convert sugar into alcohol during wine fermentation. *Saccharomyces cerevisiae* is found in vineyards, but is also found in association with oak trees and other natural sources. Although wild strains of *S. cerevisiae* as well as other *Saccharomyces* species are also capable of wine fermentation, a genetically distinct group of *S. cerevisiae* strains is primarily used to produce wine, consistent with the idea that wine making strains have been domesticated for wine production. In this study, we demonstrate that humans can distinguish between wines produced using wine strains and wild strains of *S. cerevisiae* as well as its sibling species, *Saccharomyces paradoxus.* Wine strains produced wine with fruity and floral characteristics, whereas wild strains produced wine with earthy and sulfurous characteristics. The differences that we observe between wine and wild strains provides further evidence that wine strains have evolved phenotypes that are distinct from their wild ancestors and relevant to their use in wine production.

## Introduction

Fermentation of sugars derived from fruits and starchy vegetables for the production of alcoholic beverages permeates cultures worldwide. Whether for ceremonial, religious, food safety, or nutritional reasons, the production of alcohol is embedded in human history (McGovern, 2003). The earliest evidence for wine fermentation comes from the molecular analysis of pottery jars that have been dated as far back as 7000 BC (McGovern *et al*., 2004), and extraction of DNA from ancient wine containers is consistent with the presence of the budding yeast *Saccharomyces cerevisiae* (Cavalieri *et al*., 2003). The use of *S. cerevisiae* for wine production is likely to have occurred for thousands of years and to have preceded its use for bread and beer (Mortimer, 2000; McGovern, 2003). Although *S. cerevisiae* is the dominant species used for wine, beer and bread production worldwide (Mortimer, 2000), other *Saccharomyces* species have similar fermentative capabilities, but are not as commonly used. For example, two closely related species, *Saccharomyces bayanus* and *Saccharomyces paradoxus*, are occasionally associated with wine production (Naumov *et al*., 2000, 2002; Redzepovic *et al*., 2002). In addition, *Saccharomyces pastorianus*, a hybrid between *S. cerevisiae* and *S. bayanus*, is used for lager beer fermentation (Nguyen & Gaillardin, 2005), and a number of other naturally occurring *Saccharomyces* hybrids have been associated with fermentations (Groth *et al*., 1999; de Barros Lopes *et al*., 2002; Naumova *et al*., 2005; González *et al*., 2006; Lopandic *et al*., 2007).

Wild strains of *S. cerevisiae* have been isolated from a variety of natural sources and have been frequently found in association with oak tree exudates, bark and soil (Naumov *et al*., 1998; Sniegowski *et al*., 2002). In comparison, *S. paradoxus*, the sibling species of *S. cerevisiae*, is rarely found in association with vineyards, but is frequently found in association with oak trees (Naumov *et al*., 1997, 1998; Redzepovic *et al*., 2002; Sniegowski *et al*., 2002; Johnson *et al*., 2004; Yurkov, 2005; Koufopanou *et al*. 2006; Glushakova *et al*., 2007). A number of other *Saccharomyces* species have also been found in association with oak trees and soil, and in some instances occur in sympatry with *S. cerevisiae* and *S. paradoxus* (Naumov *et al*., 1998, 2003; Sniegowski *et al*., 2002; Sampaio & Goncalves, 2008).

Strains of *S. cerevisiae* collected from ecologically and geographically diverse sources typically demonstrate genetic divergence associated with habitat type rather than geographic origin (Fay & Benavides, 2005; Legras *et al*., 2007; Borneman *et al*., 2008; Liti *et al*., 2009; Novo *et al*., 2009; Schacherer *et al*., 2009). Strains of *S. cerevisiae* associated with vineyards and wine production, hereafter referred to as ‘wine’ strains, often form a genetically differentiated group that is separate from ‘wild’ strains isolated from soil and oak tree habitats, and strains from other fermentations, such as palm wine and sake (Fay & Benavides, 2005; Legras *et al*., 2007; Liti *et al*., 2009; Schacherer *et al*. 2009; Goddard *et al*., 2010). The genetic divergence between wine and non-wine strains combined with an observed reduction in genetic diversity within wine strains suggests that wine strains were domesticated from wild *S. cerevisiae* (Fay & Benavides, 2005).

In domesticated plants and animals, a ‘domestication syndrome’ is typically present, consisting of a suite of phenotypic traits that have diverged between the domesticate and the wild ancestor (Doebley *et al*., 2006). These traits are often under strong selection themselves, or linked to traits that are under strong selection. In *S. cerevisiae*, there is evidence that phenotypic divergence has accompanied genetic divergence between wine and non-wine strains. Divergent phenotypes include resistance to copper (Fay *et al*., 2004; Liti *et al*., 2009) and sulfite (Park & Bakalinsky, 2000), two chemicals related to vineyards and wine production, as well as growth and fermentation parameters, (Spor *et al*., 2009), freeze/thaw tolerance (Will *et al*., 2010), and sporulation efficiency (Gerke *et al*., 2006).

Domestication phenotypes in *S. cerevisiae* may include wine aroma and flavor, which have been of long-standing interest to winemakers. Yeast metabolites are known to influence the sensory attributes of wine through the production of esters, higher alcohols, carbonyl compounds, volatile acids, volatile phenols, and sulfur compounds (Swiegers & Pretorius, 2005). In some cases, it has also been shown that humans can differentiate between wines fermented using different strains of *S. cerevisiae* (Wondra & Berovic, 2001; Carrau *et al*., 2008; Molina *et al*., 2009; Swiegers *et al*., 2009; Callejon *et al*., 2010). Apart from the influence of grapes and fermentation conditions, different wine yeasts also affect the flavor profile because they vary in their production of flavor-active metabolites (Herjavec *et al*., 2003; Verstrepen *et al*., 2003a, b; Estevez *et al*., 2004; Howell *et al*., 2004; Masneuf-Pomarède *et al*. 2006; Loscos *et al*., 2007; Carrau *et al*., 2008; Barbosa *et al*., 2009; Mendes-Ferreira *et al*., 2009; Molina *et al*. 2009; Swiegers *et al*., 2009; Vilela-Moura *et al*., 2010). Although the contribution of wild *S. cerevisiae* strains to wine aroma and flavor is largely unknown, studies of indigenous vineyard strains of *S. cerevisiae* have revealed variation in their production of wine aroma and flavor metabolites (Wondra & Berovic, 2001; Nurgel *et al*. 2002; Romano *et al*., 2003; Callejon *et al*., 2010; Orlic *et al*., 2010).

In this study, we investigated wine aroma and flavor using sensory and chemical analysis of grape wines fermented using wine and wild *S. cerevisiae* strains. Our results indicate that humans can distinguish between wines fermented using different wild yeast strains, and demonstrate that wine strains produce wines that are perceived as fruity and floral, whereas wild strains produced wines that are perceived as earthy and sulfurous.

## Materials and methods

### Yeast strains and fermentation

**Fig. 1:**
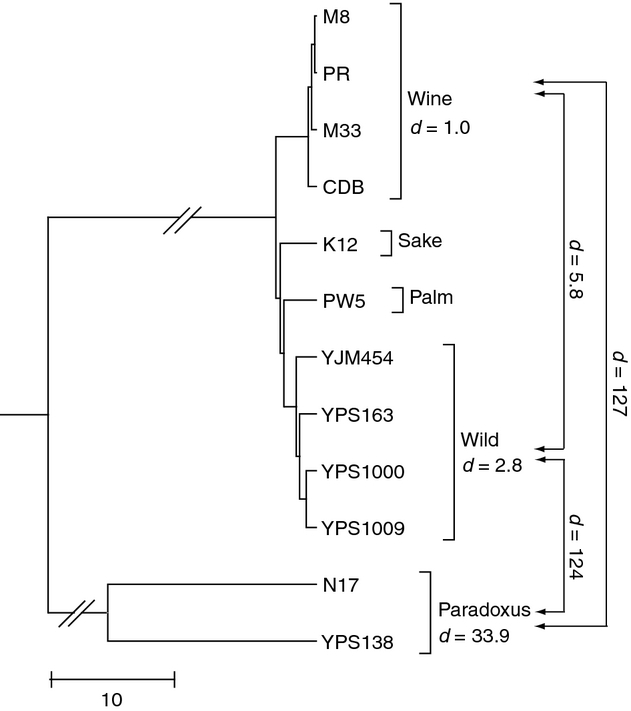
Evolutionary relationship of strains used in this study. Distance tree (upgma) based on 4379 bp at five loci, using pairwise elimination of gaps and missing data. Distances (d) are the proportion of nucleotide differences* 1000. Average pairwise distances within groups are shown where applicable, distances between groups are indicated with arrows.

The *S. cerevisiae* and *S. paradoxus* strains used in this study are described in Table 1. Strains W303, N17, and YPS138 were kindly provided by Ed Louis and Gianni Liti (Liti *et al*., 2009). Strain PW5 (NPA07) was kindly provided by O. Ezeronye (Ezeronye & Legras, 2009), and the remainder were described in a previous study (Fay & Benavides, 2005). All strains were diploid and potentially heterozygous, i.e. monosporic clones were not generated. Evolutionary relationships between strains, inferred using the upgma method based on pairwise nucleotide *P*-values at five loci, described in Fay & Benavides (2005), as implemented in mega3 (Tamura *et al*., 2007) are shown in Fig. 1. Sequences for strain PW5 were obtained by blasting whole genome assemblies available at http://www. genetics.wustl.edu/jflab/data3.html, sequences for strains W303, N17, and YPS138 were obtained by blasting whole genome sequences available at http://www.sanger.ac.uk/research/projects/genomeinformatics/sgrp.html, and the remainder were described in a previous study (Fay & Benavides, 2005).

**Table 1:** Yeast strains used in this study

Strain	Class	Origin
W303	Laboratory	Related to the laboratory strain S288c
Cotes des Blancs (CDB)	Grape wine	Commercial wine strain originating from Germany
Pasteur Red (PR)	Grape wine	Commercial wine strain originating from France
M33	Grape wine	Vineyard, Italy
M8	Grape wine	Vineyard, Italy, 1993
YPS163	Wild	Oak exudate, Pennsylvania, United States, 1999
YPS1000	Wild	Oak exudate, New Jersey, United States, 2000
YPS1009	Wild	Oak exudate, New Jersey, United States, 2000
YJM454	Wild	Clinical isolate (blood), United States, pre-1994
PWB	Palm wine	Raphia Palm tree, Aba, Abia state, Nigeria, 2002
AKU-4011 (K12)	Sake	Commercial Sake wine, Japan
N17	*S. paradoxus*	Oak exudate, Tartarstan, Russia
YPS138	*S. paradoxus*	Oak soil, Pennsylvania, United States, 1999

Fermentations were conducted using sterile concentrated grape juice from Vintners Reserve Chardonnay kits (Winexpert, Port Coquitlam, BC, Canada). Juice was distributed into sterilized two gallon food grade plastic buckets fitted with airlocks for primary fermentation. Yeast starter cultures were grown individually in 150 mL of sterile juice and used to inoculate 1.25 gallons of juice at a density of 2–5 9 10 cells mL”. When specific gravity reached 1.010, juice was transferred into 1 gallon glass carboys for secondary fermentation. After fermentation was complete, as measured by absence of CO_2_ release and glucose concentrations <0.5%, the wine was stabilized using metabisulphite and sorbate, cleared with isinglass, and bottled in 375 or 750 mL glass wine bottles with synthetic cork closures. Between two and ten replicate wine fermentations were generated per strain.

### Discriminatory sensory evaluation

Discriminatory sensory evaluation was performed by the use of triangle tests (Stone & Sidel, 2004) to assess the significance of perceived sensory differences between wines. Trays with three samples of wine were served to participants. Two of the samples on each tray were identical, whereas one was different. Samples were labeled with a randomized three digit number, and participants were asked to circle the number corresponding to the sample that was different. Four to six trays (triangles) were served to each participant during each session. Tests were performed using a balanced block design: triangles, serving orders, and positions were balanced to allow for the detection of positional effects.

To test the validity of the method and the performance of participants, we carried out an initial discrimination test using a wine strain (CDB) and a lab strain (W303) (experiment A, Table 2). Sixty-five participants evaluated this triangle six times each *(N =* 390). Participants distinguished between these two wines 42% of the time, significantly more often than the 33% expected by chance (Binomial test, *P <* 0.001). The distribution of the participants’ individual scores approximated the binomial distribution, suggesting that judges were equally skilled at detecting differences. Power analysis was used to determine that 100 evaluations were needed to detect differences. We found no significant difference between serving order (e.g. tray one through six), triangle (e.g. two CDB with one W303 or *vice versa)*, position of the outlier on the tray (e.g. outlier in the first, second or third position left to right), fermentation replicate, or bottle using a Chi-square test. These effects were also not significant during any discriminatory evaluations, with the following exception: during experiment B (wine and wild *S. cerevisiae* compared to *S. paradoxus)*, the proportion of correct decisions for the second and fourth trays were significantly different (Chi-square, *P =* 0.003). To test for outliers, each strain was compared with the rest of the strains within the same class (e.g. wine, wild, and *S. paradoxus)* using a Chi-square test.

**Table 2:** Human discrimination of wines produced by different strains of *Saccharomyces cerevisiae*

Experiment	Comparison	Proportion correct*	SE	Triangle tests	*P*†	Judges‡	Trays perjudge§
A	Between wine (CDB) and lab (W303)	0.42	0.02	390	< 0.001	656	6
B	Between wine (CDB) and paradoxus (N17, YPS138)	0.46	0.05	96	0.007	54	4
B	Between wild (YPS163) and paradoxus (N17, YPS138)	0.45	0.05	96	0.004	54	4
B	Between wine (CDB) and wild (YPS163)	0.43	0.05	96	0.021	54	4
C	Within wild (YPS163, YPS1009, YPS1000, YJM454)	0.40^A^	0.04	190	0.023	51	6
C	Within wine (CDB, M33, M8, PR)	0.47^A^	0.04	190	< 0.001	51	6
C	Between wine (CDB, M33, M8, PR) and wild (YPS163, YPS1009, YPS1000, YJM454)	0.56^B^	0.04	190	< 0.001	51	6
D	Between palm (PW5) and wild (YPS1000, YPS1009)	0.36	0.08	39	0.301	52	5
D	Between palm (PW5) and wine (CDB, M8)	0.52	0.07	48	0.002	52	5
D	Between sake (K12) and wild (YPS1000, YPS1009)	0.00	0.07	48	0.006	52	5
D	Between sake (K12) and wine (CDB, M8)	0.42	0.07	48	0.086	52	5
D	Between palm (PW5) and sake (K12)	0.46	0.10	24	0.068	52	5

* Superscript letters in the proportion correct column indicate significance group differences between comparisons within an experiment (*P* < 0.05, Chi-square test).

† *P*-values are calculated using the binomial test as deviation from random expectation (proportion correct = 0.33).

‡ Judges indicates the number of individuals who participated in each experiment.

§ Trays per judge indicate the number of triangle tests performed by an individual in a single session. Two sessions were performed over 2 days for each experiment.

### Quantitative descriptive sensory analysis

A preliminary flavor/taste development session was conducted by Vinquiry, Inc (Sonoma, CA) using six wine experts to evaluate a subset of the wines for aroma and flavor (W303, YPS1000, PW5, N17, K12, and CDB). From this evaluation, 28 aromas and five flavors, representing eleven classes from the wine aroma wheel were found including: chemical, pungent, floral, fruity, vegetative, caramelized, woody, earthy, microbiological, oxidized, and nutty. The results were filtered according to the number of wines in which the attribute was present, the number of panelists who reported the attribute for a given wine, and to ensure adequate representation of different classes of aroma and flavor. On the basis of these criteria, we chose 12 attributes for descriptive analysis: cabbage (sulfur), wet dog (sulfur), floral, citrus (fruity), tree fruit (fruity), oxidized (acetaldehyde), hay/ straw (vegetative), mushroom (earthy), butterscotch (caramel), acidity (taste), astringency (taste), and trueness to style (taste and aroma). Style trueness was measured relative to a traditional, commercial un-oaked chardonnay. A quantitative descriptive analysis of all 12 attributes was conducted for each wine using an independent panel of six expert judges. Judges underwent three training sessions to review properties of aroma and taste identification as well as variation in aroma/flavor intensity using standard references. Judges scored aroma/flavor attributes based on a numerical scale of 0–9 in duplicate for each wine.

Statistical analysis of wine characteristics was carried out using r (R Development Core Team, 2009). Each judge's scores for each attribute were centered on the judge's mean score for that attribute and scaled to a standard deviation of 1. Principal component analysis (PCA) and linear descriptive function analysis (LDA) was performed on the transformed data. A stepwise selection criterion was employed to determine which combination of attributes optimized the predictive value for grape wine, oak, and *S. paradoxus* strains. Analysis of variance (anova) was conducted on the values for the first two principal components as well as on the transformed scores for each of the 12 aroma/flavor attributes with the model. For attributes that were significantly different for the class or strain term using univariate anova, a *post-hoc* Tukey's honestly significant difference (HSD) test was performed to determine which classes and/or strains were significantly different from each other. No significant effects were found for tasting session or wine replicate using anova. For all anovas, the normality of the residual distribution was examined using the Shapiro–Wilk's normality test. When residuals were not normally distributed, data transformations were applied as determined using a Box–Cox power transformation. The following transformations were applied: for the first principal component scores *y = x* + 10^−0.8383^, for oxidized *y = x* + 10^−0.8686^, for tree fruit *y* = *x* + 10^−1.0303^, and for citrus *y = x* + 10^−1.4747^. No suitable data transformations were found for butterscotch, trueness to type, or floral. For those attributes, permutation tests (*N* = 10 000) were used to generate an empirical F distribution and determine the probability of the observed mean differences between classes and strains. Empirical *P*-values were corrected for multiple testing using the Bonferonni method. Pearson's r rank correlation coefficients were calculated for all possible pairs of attributes.

### Chemical analysis

Chemical analyses were carried out to determine the concentration of the given chemicals in a sample of wine from each of the wine, oak, and *S. paradoxus* strains listed in Table 1. A basic chemistry panel (free sulfur dioxide, molecular sulfur dioxide, total sulfur dioxide, titratable acidity, pH, and volatile acidity), higher alcohol and fusel oil panel (acetaldehyde, ethyl acetate, methanol, 1-propanol, iso butanol, A-amyl alcohol, and I-amyl alcohol), and sulfides panel (hydrogen sulfide, methyl mercaptan, ethyl mercaptan, dimethyl sulfide, dimethyl disulfide, diethyl sulfide, diethyl disulfide) was performed by ETS Laboratories (St Helena, CA). In addition, acetaldehyde, ethyl acetate, ethyl propionate, ethyl isobutyrate, isobutyl acetate, ethyl butyrate, propanol, ethyl-2-methylbutyrate, ethyl-3-methylbutyrate, isobutanol, isoamyl acetate, butanol, isoamyl alcohl, ethyl hexanoate, ethyl octanoate, and phenyl ethanol were measured using headspace gas chromatography coupled with flame ionization detection (GC-FID). Samples were analyzed with a calibrated HP 6890 Series GC System (Agilent Technologies, Santa Clara, CA) with a headspace sampler (PAL system; CTC Analytics, Zwingen, Switzerland) and equipped with a DB-WAXETR column (length, 30 m; internal diameter, 0.25 mm; layer thickness, 0.5 lm; Agilent Technologies). Analyses were carried out in duplicate, and the results were analyzed with Chemstation (Agilent Technologies). Correlations between measurements of ethyl acetate, propanol, isobutanol, and isoamyl alcohol were all >0.90 for the two data sets, and 0.61 between acetaldehyde measurements. ETS generated data for these chemicals were removed from the dataset for analysis. Statistical analysis of chemical concentrations was carried out using r (R Development Core Team, 2009). Individual anovas were performed on each chemical to test for significant differences between classes (wine, wild, and paradoxus). LDA was performed to determine the predictive power of the chemical composition of wines fermented with grape wine, oak, and *S. paradoxus* strains, and a stepwise selection criterion was employed to determine which combination of attributes optimized the predictive value. Pearson's r rank correlation coefficients were calculated for all possible pairs of chemicals, and for all possible pairs of descriptive attributes and chemicals.

## Results

### Human discrimination of wines fermented using wine yeast and non-wine yeast

A series of triangle discrimination tests were used to determine the ability of humans to discriminate between wines fermented using different yeast strains (see Table 1 for a description of strains, and Table 2 for a description of experiments). In the discrimination test, participants were presented with three samples of wine, two of which were fermented using the same strain and one of which was fermented using a different strain.

We hypothesized that humans can discriminate between wines fermented using strains of the same class (i.e. wine or wild) significantly more often than random, and that humans can discriminate between wines fermented using wine strains and those fermented using wild strains significantly more often than when presented with wines fermented using two different strains of the same class (i.e. wine or wild). To test these hypotheses, we measured rates of discrimination between all pairwise combinations of four wines produced with wine strains (CDB, PR, M33, and M8) and four wines produced using wild strains (YPS163, YPS1000, YPS1009, and YJM454), both within and between each group, using the triangle test (experiment C, Table 2). For each type of comparison, the proportion of correct classifications was significantly higher than 33%, the proportion expected by chance, indicating that humans can distinguish between wines produced by different strains regardless of their class, and establishing human perception as a selectable yeast phenotype. The ability of participants to discriminate between wines produced by wild strains was the lowest at 40% (Binomial test, *P =* 0.023), followed by wine strains at 47% *(P <* 0.001), and was highest between wine and wild strains at 56% *(P <* 0.001) (experiment C, Table 2). No single comparison showed evidence of being an outlier based on the number of correct and incorrect decisions for each comparison (within wine, within wild, and between wine and wild). The magnitude of discrimination (47%) between wine strains was not significantly different from the magnitude of discrimination between wild strains (40%). However, discrimination between wine and wild strains (56%) was significantly greater than that within either group (Chi-square test, *P =* 0.040, and *P =* 0.001 for comparisons between wine and wild strains to those within wine and within wild, respectively (experiment C, Table 2).

A separate discrimination experiment (experiment B, Table 2) was performed to measure the ability of humans to discriminate wines fermented using two *S. paradoxus* strains (N17 and YPS138) with wines fermented using a randomly selected grape wine strain (CDB) and a randomly selected wild strain (YPS163) of *S. cerevisiae*. We measured the ability of participants to discriminate between wines fermented using the wine and wild *S. cerevisiae* strains as well as their ability to discriminate between wines fermented using each *S. cerevisiae* strain and each of the two different *S. paradoxus* strains. Strikingly, the wines fermented using wine and wild strains were as distinguishable from each other as either was from wines fermented using *S. paradoxus* (Table 2). The ability of humans to discriminate between wines fermented using *S. cerevisiae* strains and *S. paradoxus* was not significantly different for either strain of *S. paradoxus*. In addition, pairwise discrimination between wine and wild for CDB and YPS163 (46%) was not significantly different from the same comparison made in experiment C.

Although most strains of *S. cerevisiae* have been found in association with vineyards and oak trees, strains have also been found in association with other wine fermentations, including sake and palm wine. To determine whether human perceived differences between wines fermented using wine and wild strains is associated with historic use for the production of alcoholic beverages, we measured the ability of participants to discriminate between wines fermented using either a palm wine (PW5), sake (K12), two randomly chosen grape wine (CDB or M8), or two randomly chosen wild (YPS100 or YPS1009) strains of *S. cerevisiae*. Subjects were unable to distinguish between wine fermented using the palm strain and wild strains, but were able to distinguish wine fermented using the palm strain and wine strains (experiment D, Table 2). The ability of participants to distinguish between wine fermented using the palm strain and the wine strains was similar to the degree of differentiation observed when subjects discriminated between wines fermented using grape wine and wild strains (experiment D, Table 2). In contrast, the wine fermented using the sake strain was significantly different from that fermented using the wild strains, but not significantly different from the wines fermented using wine and palm strains.

### Quantification of sensory attributes

The results of our discrimination tests demonstrate that *S. cerevisiae* strains produce wines that can be discriminated by human perception. However, discrimination testing does not allow us to quantify differentiation for specific attributes. To determine which sensory attributes contribute to the perceived sensory differences between wines fermented using different strains, the same wines used in our discriminatory analysis were used for quantitative descriptive analysis (see Table 1 for a description of strains). As described in Materials and methods, 12 attributes (cabbage, wet dog, oxidized, mushroom, astringency, acidity, hay/straw, butterscotch, tree fruit, trueness to type, citrus, and floral) were chosen for analysis and a trained panel of experts evaluated each wine for those 12 attributes using a quantitative scale.

**Fig. 2:**
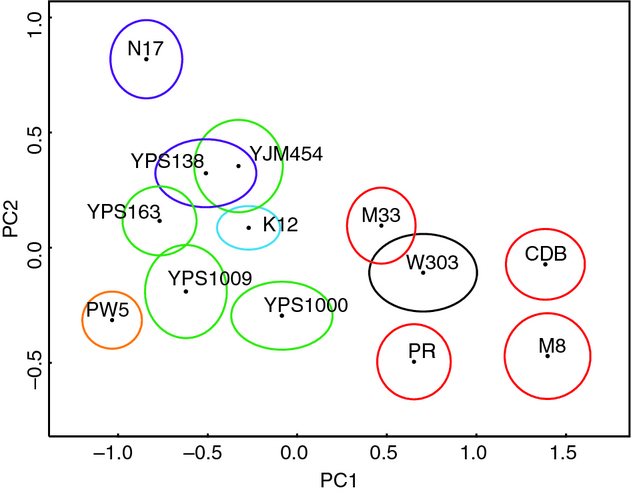
Wine and non-wine strains of *Saccharomyces cerevisiae* are differentiated for wine flavor and aroma attributes. Strain means (points) and standard error (ellipses) of the first two principal components for 12 wine attributes measured using quantitative descriptive analysis.

PCA was used to evaluate differences in scores for the 12 attributes. The first two principal components together explained 35.4% of the variance. The mean and standard error of the first two principal components for each strain is shown in Fig. 2. The first principal component axis, which explains 23.7% of the variation, was loaded most heavily by cabbage, wet dog, oxidized and mushroom attributes in the negative direction, and by butterscotch, tree fruit, trueness to type, citrus, and floral attributes in the positive direction (Table S1). The grape wine strains along with the lab strain W303, which is closely related (genetically) to wine strains (Rothstein, 1977; Rothstein *et at*, 1977; Winzeler *et al*., 2003), are associated with positive values on the first principal component axis, whereas wild, palm, sake, and *S. paradoxus* strains are associated with negative values on this axis. The second principal component axis, which explains 11.7% of the variation, was loaded most heavily by astringency, acidity, wet dog, floral, and cabbage attributes in the negative direction and by butterscotch, hay/straw, and mushroom attributes in the positive direction (Fig. 2). Significant correlations were found between sensory attributes, supporting the relationships inferred through PCA (Table S5). Linear discriminant analysis (LDA) was performed to determine the predictive value of the 12 attributes. Overall, the linear descriptive analysis was able to correctly classify 51% of observations (67% for oak, 65% for grape wine, 36% for sake, 33% for paradoxus, 27% for lab, and 9% for palm wine strains). When considering grape wine, wild, and *S. paradoxus* strains alone, in agreement with PCA, the combination of variables that optimized the predictive value included wet dog, citrus, and floral (67% for grape wine, 70% for wild, and 25% for *S. paradoxus.)*

To determine if there was a significant difference between classes of strains for principal components, we performed anova on the principal components scores for each axis, as described in Materials and methods. The class term, with wine, wild, palm, sake, and *S. paradoxus*, was significant for the first principal component *(P <* 0.001), but not for the second principal component *(P =* 0.124). The strain term, which represents random strain effect within each class, was not significant for either of the first two principal components *(P =* 0. 816 and *P =* 0.591, respectively) (Table S1). A *post-hoc* Tukey's HSD test revealed that wines fermented using grape wine strains are significantly different from those fermented using wild, palm wine, and *S. paradoxus* strains for the first principal component (Table S2), but not significantly different from sake or lab strains. Despite some levels of discrimination between sake, palm, wild, and *S. paradoxus* strains (experiment C, Tables 2 and S2), these classes are not significantly different from one another for wine attributes captured by the first principle component, which readily distinguishes grape wine strains from other strains of *S. cerevisiae* and *S. paradoxus*. Similarly, linear discriminate analysis is able to predict class membership for each wine replicate 65% and 67% of the time for wine and wild strains, respectively, but only 27% of the time, on average, for the other classes.

**Fig. 3:**
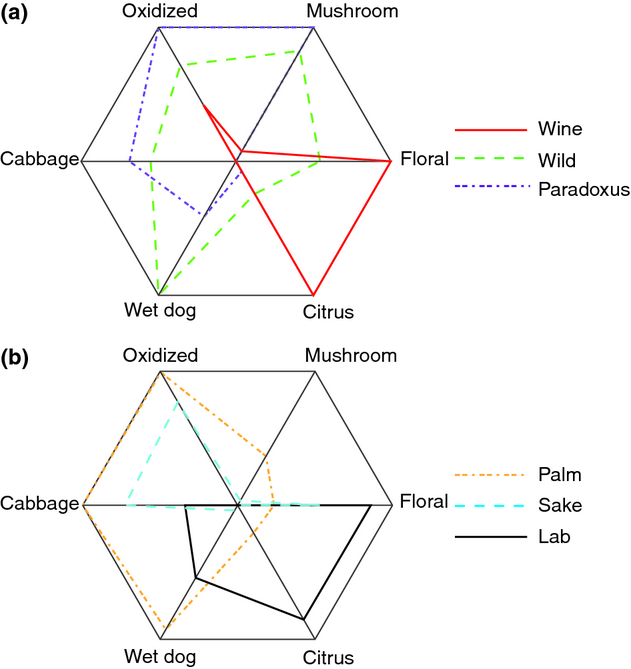
Sensory attributes differentiate between wine and non-wine strains of *Saccharomyces cerevisiae*. (a) Class means for wine strains, wild strains, and *Saccharomyces paradoxus* strains, and (b) means for the palm, sake, and laboratory strains are shown for each of the six quantitative descriptive wine attributes that distinguish wine strains from other non-wine strains. Means were scaled from 0 (center) to 1 (spokes), where 0 represents the lowest mean score, and 1 represents the highest mean score for any class.

In agreement with the PCA analysis, wine attributes that are significantly different between classes by anova include cabbage, wet dog, oxidized mushroom, citrus, and floral (Table S1). Differences in the mean class scores for these attributes are depicted in Fig. 3. Wines fermented using wild, palm, sake, and *S. paradoxus* strains scored higher for undesirable attributes, whereas wines fermented using grape wine strains and the lab strain scored higher for desirable attributes. *Post-hoc* Tukey's HSD tests revealed that cabbage, wet dog, citrus, and floral attributes differentiated between grape wine strains and other strains, but not between any classes of non-grape wine strains (Table S2). Mushroom aroma was variable between many classes, differentiating grape wine strains from wild *S. cerevisiae* and *S. paradoxus* strains, but also differentiating sake strains from wild *S. cerevisiae* and *S. paradoxus* strains (Table S2). Oxidized aroma did not differentiate wine strains from any other class of strains (Table S2). The only significant differences between strains within a class was between two grape wine strains, M33 and CDB *(P =* 0.044) for mushroom aroma. The results of this quantitative analysis support our results of the initial discrimination tests, showing that human perceived differences between wines produced by wine strains and other classes of strains are significantly greater than differences within each class. In addition, the aromas that contributed the most to the perceived differences between wine and wild strains are cabbage, wet dog, citrus and floral, with wine yeasts being associated with the latter two.

### Chemical analysis

A final experiment was conducted to test if the flavor and aroma attributes that contribute to the ability of humans to discriminate between wines fermented using wine strains and those fermented using wild strains and *S. paradoxus* strains are due to differences in chemical concentrations produced during fermentation. The chemical composition of the wines was evaluated for 25 chemicals, including commonly produced yeast metabolites associated with wine flavor (Table S6). Overall, the chemical composition was able to predict the class of the wine 90% of the time (75% for oak, 100% for *S. paradoxus*, and 100% for grape wine strains). The combination of propanol (alcohol aroma), ethyl octanoate (green apple aroma), and ethyl propionate (plum, apple aroma) alone was able to completely distinguish between classes. Each chemical attribute was also considered independently using anova (Table S3), and a *post-hoc* Tukey's HSD for chemical attributes that were significantly different between classes by anova (Table S4). Wine strains produced significantly more propanol than oak strains (Tukey's HSD *P =* 0.024), and *S. paradoxus* strains produce significantly less ethyl octanoate than wine or oak strains (Tukey's HSD *P =* 0.001 for each comparison). In addition, *S. paradoxus* strains produced significantly more ethyl-2-methylbutyrate (fruity, apple aroma) than wine strains *(P =* 0.019), significantly more butanol (alcohol aroma) than wine or oak strains *(P =* 0.006 and *P =* 0.009, respectively), and significantly less isoamyl acetate (banana, pear aroma) than wine or oak strains (P = 0.046 and *P =* 0.015, respectively). Similar to descriptive attributes, significant correlations were found between chemicals, and also between chemicals and descriptive attributes (Table S5).

## Discussion

Many *Saccharomyces* yeasts preferentially ferment sugar into alcohol in the presence of oxygen despite the higher energy yield of respiration (de Deken, 1966). However, grape wine is often produced using a genetically homogeneous subgroup of *S. cerevisiae* strains (Fay & Benavides, 2005; Legras *et al*., 2007; Liti *et al*., 2009; Schacherer *et al*., 2009), thought to have been domesticated for wine-making. The reduced levels of variation present in wine-making strains could have been the result of a genetic bottleneck, selection for specific traits, or a combination of the two. In the case of domestication, it is expected that differentiation of certain phenotypic traits (domestication phenotypes) will accompany genetic differentiation. Herein, we investigate wine aroma and flavor as a potential domestication phenotype.

Our results demonstrate that humans can differentiate between wines fermented using different strains of yeast, regardless of the strain's origin. We also demonstrate that divergence in wine aroma and flavor is coupled with the genetic divergence between wine and wild strains, consistent with the hypothesis that wine aroma and flavor is a domestication phenotype. Furthermore, the magnitude of phenotypic divergence between grape wine and wild strains of *S. cerevisiae* compared with *S. paradoxus*, suggests rapid enological divergence of the wine strains from their wild ancestors.

Wine and non-wine strains are differentiated by several sensory attributes. We found that the sulfurous attributes, cabbage and wet dog, make a major contribution to differences between wines produced by wine strains and those produced by wild strains of *S. cerevisiae* and strains of *S. paradoxus*. Citrus and floral attributes make similar contributions to the difference between wine and wild *S. cerevisiae* strains. However, it is important to note the possibility that citrus and floral attributes were present in wines produced by wild strains at levels similar to those produced by wine strains, but were detected at a lower level by humans due to the masking effect of sulfurous attributes. Other attributes that contributed to the difference between wine and wild strains included the oxidized aroma and mushroom aroma, but the latter was more heavily loaded on the second principle component. Although the second principal component was not significantly different among classes of yeast strains, it tended to differentiate wild strains of *S. cerevisiae* and strains of *S. paradoxus*. The attributes astringency, acidity, hay/ straw, and butterscotch were also more heavily loaded on the second principle component axis, but did not make significant contributions to differences between classes of yeast strains. The variation in sulfur-related attributes may be important for the contemporary wine industry as the production of hydrogen sulfide, thiols (mercaptans), and related sulfur-containing compounds during fermentation is a major problem in wine production (Swiegers & Pretorius, 2007). Commercial wine strains of *S. cerevisiae* (Swiegers & Pretorius, 2007), as well as *S. bay anus* (Ugliano *et al*., 2009) differ in their production of sulfur compounds, which is often influenced by fermentation conditions and grape juice composition.

We found the largest differences in perceived wine aroma between wines produced by wine and wild strains of *S. cerevisiae*, which was equal to the differences between wine strains of *S. cerevisiae* and strains of *S. paradoxus*. This degree of phenotypic divergence within *S. cerevisiae* groups (wine vs. wild yeasts) is remarkably high, given that the genetic divergence between *S. cerevisiae* and *S. paradoxus* is 25 times higher than that between a wine and wild strain of *S. cerevisiae* (Doniger *et al*., 2008). Enological divergence among wine strains was similar to that among wild strains, despite the latter showing 3.6 times more genetic diversity (Fay & Benavides, 2005). However, this pattern is consistent with previous studies that revealed substantial variation in stress response (Kvitek *et al*., 2008) and growth and fermentation parameters (Spor *et al*., 2009) among wine strains compared with other *S. cerevisiae* strains. In addition, the increased phenotypic diversity combined with a reduction in genetic diversity is consistent with other domesticated organisms (e.g. varietal differences in crops (Doebley *et al*., 2006)).

The smaller enological differences between the sake, palm wine, and wild strains is not surprising. The attributes that were dominant in *S. paradoxus* were also dominant in wild *S. cerevisiae* strains. The lack of phenotypic divergence between wild *S. cerevisiae* and *S. paradoxus* strains might be a consequence of constraints placed upon them by their shared environment. Palm wine strains produce wine with attributes that are similar to wild strains, and sake strains produce wine with attributes intermediate between wine and wild strains. However, the low levels of differentiation among these groups could be due to the measurement of grape wine attributes rather than sake or palm wine attributes. Consistent with this possibility, sake strains exhibit a number of sake fermentation flavor characteristics that differ from those generated by a laboratory strain (Katou *et al*., 2008, 2009). Thus, differentiation between wine and wild strains does not appear to be simply correlated with use in alcohol production.

One limitation of our study is the small number of strains analyzed in each group and the fermentation of only a single grape juice. Although it is hard to know whether the strains used in our study are representative of the phenotypic diversity present in other wine and wild yeast strains, the strains were selected based on sequence variation present in five genes (Fig. 1) and so no phenotypic bias is expected. Interestingly, genotypically wild strains of *S. cerevisiae* have been isolated from fermenting grape musts in New Zealand (Goddard *et al*., 2010). This raises the question of what impact wild yeast have on mixed fermentations. Further research will be needed to establish the full extent of variation in wine aroma and flavor phenotypes generated by different *S. cerevisiae* strains and under different fermentation conditions. One approach supported by our work is to use wine metabolites to characterize variation in wine aroma and flavor.

Measurement of chemical differences among wines revealed a number of quantitative differences, including some that significantly differentiated wine and wild strains. Several of the chemicals that were found to discriminate between wine, wild, and *S. paradoxus*, strains were significantly correlated *(P <* 0.05) with descriptive attributes that also discriminated between these types of strains, indicating that the differences in descriptive sensory profiles are likely to correspond to differences in the chemical profile of these wines. Most notably, increased levels of propanol and ethyl-2-methylbutyrate were negatively correlated with wet dog and citrus aromas, respectively, and ethyl octanoate was positively correlated with floral aroma. However, hundreds of compounds are known to influence wine flavor and aroma (Swiegers & Pretorius, 2005), many of which could contribute to attributes that distinguish wine and wild strains. Moreover, the overall aroma of a beverage is the result of subtle combinations of various chemical compounds, and small changes in one or a few compounds can have profound and unpredictable effects on the overall aroma. Determining the genetic contribution of *S. cerevisiae* to wine flavor and aroma characteristics is challenging (Bisson & Karpel, 2010). Not only do yeast metabolites interact to form certain flavors and aromas, but grape composition and fermentation conditions affect *S. cerevisiae* metabolite production (Bisson & Karpel, 2010). Despite this difficulty, several examples of genes underlying wine flavor and aroma differences have been identified. Genes involved in the production of fusel oils (higher alcohols), volatile organic acids, esters, sulfur-containing volatiles, carbonyl compounds, volatile aglycones, and cys-conjugates have been identified (Verstrepen et al., 2003a, b; Howell *et al*., 2005; Lilly *et al*., 2006; Saerens *et al*., 2006; Bisson & Karpel, 2010). Genetic variation at these loci between wine and wild strains of *S. cerevisiae* may account for some of the observed differences in wine flavor and aroma, but further work will be needed to dissect the genetic basis for the sensory differentiation we observed between wine and wild strains of *S. cerevisiae*.

Although most differences in wine quality are attributable to grapes, which differ by variety, location, and year, there is a growing body of evidence that wine quality is also influenced by the yeast (Swiegers & Pretorius, 2005; Bisson & Karpel, 2010), specifically in the production of undesirable sulfur aromas (Swiegers & Pretorius, 2007; Bisson & Karpel, 2010). Our results show that wild *S. cerevisiae* may in some cases contribute several undesirable wine characteristics, resulting in low quality wine. If wild populations of *S. cerevisiae* are present in vineyards during grape harvesting, they may contribute to problem fermentations. By identifying the genetic determinants of undesirable attributes present in wild yeast populations, it may be possible to further improve existing commercial wine strains (Pretorius & Bauer, 2002) as well as better understand the origins and evolution of wine strains.

## Statement

Re-use of this article is permitted in accordance with the Terms and Conditions set out at http://wileyonlinelibrary. com/onlineopen#OnlineOpen_Terms

## Supporting Information

**Table S1.** PCA loadings and anova *P*-values for individual descriptive attributes.

**Table S2.** Tukey's honestly significant difference (HSD) *P*-values for descriptive attributes.

**Table S3.** anova *P*-values for individual chemical attributes.

**Table S4.** Tukey's honestly significant difference (HSD) *P*-values for chemical attributes.

**Table S5.** Significant correlations between descriptive and chemical attributes.

**Table S6.** The concentration of chemical compounds in wine produced by oak, palm, paradoxus, sake, wine, and lab strains of *S. cerevisiae*.
