# Supplementary material for: Divergence in wine characteristics produced by wild and domesticated strains of Saccharomyces cerevisiae
Source: FEMS Yeast Res. 2011 Sep 2;11(7):540–51. doi: 10.1111/j.1567-1364.2011.00746.x (PMC3262967; doi:10.1111/j.1567-1364.2011.00746.x)
Supplement: Supplementary file 1 [file fyr0011-0540-SD1.docx]

Table S1. PCA loadings and ANOVA p values for individual descriptive attributes.

|  | **PCA loadings** | | **attribute p value^1^** | |
| --- | --- | --- | --- | --- |
| **attribute** | **PC1** | **PC2** | **class** | **strain** |
| cabbage | -0.435 | -0.143 | **< 0.000** | **0.013** |
| wet dog | -0.370 | -0.256 | **0.013** | 0.599 |
| oxidized | -0.311 | 0.000 | **0.006** | 0.118 |
| mushroom | -0.209 | 0.446 | **< 0.000** | **0.017** |
| astringency | 0.000 | -0.482 | 0.122 | 0.739 |
| acidity | 0.000 | -0.274 | 0.110 | **0.009** |
| hay/straw | 0.000 | 0.444 | 0.112 | 0.086 |
| butterscotch | 0.130 | 0.419 | 0.186 | 0.476 |
| tree fruit | 0.229 | 0.000 | 0.148 | 0.328 |
| trueness | 0.355 | 0.000 | 0.213 | 0.515 |
| citrus | 0.372 | 0.000 | **0.000** | 0.156 |
| floral | 0.436 | -0.152 | **0.006** | **0.016** |

^1^ p values are from ANOVA except for butterscotch and trueness to type which are from the smallest Bonferroni corrected p value for any pairwise comparison obtained from a permutation test of mean differences between classes or strains.
