# Supplementary material for: Divergence in wine characteristics produced by wild and domesticated strains of Saccharomyces cerevisiae
Source: FEMS Yeast Res. 2011 Sep 2;11(7):540–51. doi: 10.1111/j.1567-1364.2011.00746.x (PMC3262967; doi:10.1111/j.1567-1364.2011.00746.x)
Supplement: Supplementary file 2 [file fyr0011-0540-SD2.docx]

Table S2. Tukey's HSD (Honestly Significant Difference) p values for descriptive attributes.

| **Class1** | **Class2** | **PC1^1^** | **cabbage^1^** | **wet dog^1^** | **oxidized^1^** | **mushroom^1^** | **citrus^1^** | **floral^2^** |
| --- | --- | --- | --- | --- | --- | --- | --- | --- |
| grape wine | lab | 0.974 | 0.675 | 0.829 | 0.751 | 1.000 | 0.985 | 10.833 |
| grape wine | wild | **0.000** | **0.003** | **0.008** | 0.399 | **0.000** | **0.001** | 0.951 |
| grape wine | palm | **0.001** | **0.000** | 0.304 | 0.183 | 0.883 | 0.120 | 0.707 |
| wine | paradoxus | **0.000** | **0.002** | 0.840 | 0.084 | **0.000** | **0.001** | **0.006** |
| wine | sake | 0.223 | **0.021** | 1.000 | 0.701 | 1.000 | **0.012** | 3.414 |
| sake | lab | 0.844 | 0.759 | 0.947 | 0.243 | 1.000 | 0.304 | 7.686 |
| sake | wild | 0.990 | 0.971 | 0.264 | 1.000 | **0.041** | 0.975 | 14.604 |
| sake | palm | 0.731 | 0.935 | 0.613 | 0.980 | 0.921 | 0.986 | 8.373 |
| sake | paradoxus | 0.905 | 1.000 | 0.973 | 0.992 | **0.015** | 1.000 | 3.209 |
| paradoxus | lab | 0.156 | 0.732 | 1.000 | 0.028 | **0.014** | 0.190 | 0.618 |
| paradoxus | wild | 0.984 | 0.971 | 0.534 | 0.875 | 0.957 | 0.948 | 1.023 |
| paradoxus | palm | 0.991 | 0.812 | 0.897 | 1.000 | 0.256 | 0.981 | 8.759 |
| palm | lab | 0.110 | 0.207 | 0.983 | **0.048** | 0.894 | 0.705 | 3.083 |
| palm | wild | 0.858 | 0.367 | 1.000 | 0.869 | 0.522 | 1.000 | 6.509 |
| wild | lab | 0.283 | 0.939 | 0.907 | 0.123 | **0.038** | 0.425 | 6.306 |

^1^ p value from Tukey’s HSD.

^2^ Bonferroni corrected p value from a permutation test of mean differences between classes.
