# Supplementary material for: Divergence in wine characteristics produced by wild and domesticated strains of Saccharomyces cerevisiae
Source: FEMS Yeast Res. 2011 Sep 2;11(7):540–51. doi: 10.1111/j.1567-1364.2011.00746.x (PMC3262967; doi:10.1111/j.1567-1364.2011.00746.x)
Supplement: Supplementary file 3 [file fyr0011-0540-SD3.docx]

Table S3. ANOVA p values for individual chemical attributes.

| **attribute** | **degrees of freedom** | **F value** | **p value** |
| --- | --- | --- | --- |
| acetaldehyde | 2 | 0.668 | 0.543 |
| ethyl acetate | 2 | 1.667 | 0.256 |
| ethyl propionate | 2 | 0.706 | 0.526 |
| ethyl isobutyrate | 2 | 1.506 | 0.286 |
| isobutyl acetate | 2 | 1.726 | 0.246 |
| ethyl butyrate | 2 | 1.509 | 0.285 |
| propanol | 2 | 6.561 | **0.025** |
| ethyl 2 methylbutyrate | 2 | 6.809 | **0.023** |
| ethyl 3 methylbutyrate | 2 | 1.949 | 0.212 |
| isobutanol | 2 | 0.599 | 0.575 |
| isoamyl acetate | 2 | 7.604 | **0.018** |
| butanol | 2 | 11.835 | **0.006** |
| isoamyl.alcohol | 2 | 0.394 | 0.688 |
| ethyl hexanoate | 2 | 2.361 | 0.165 |
| ethyl octanoate | 2 | 22.907 | **0.001** |
| phenyl ethanol | 2 | 1.868 | 0.224 |
| free so_2_ | 2 | 0.756 | 0.504 |
| molecular so_2_ | 2 | 7.898 | 0.220 |
| total so_2_ | 2 | 1.085 | 0.389 |
| titratable acidity | 2 | 0.772 | 0.498 |
| pH | 2 | 0.924 | 0.440 |
| volatile acidity | 2 | 2.140 | 0.188 |
| methanol | 2 | 0.102 | 0.904 |
| A-amyl alcohol | 2 | 0.019 | 0.982 |
| dimethyl_sulfide | 2 | 0.320 | 0.736 |

ANOVA was calculated for classes (wine, wild and paradoxus).
