# Supplementary material for: Divergence in wine characteristics produced by wild and domesticated strains of Saccharomyces cerevisiae
Source: FEMS Yeast Res. 2011 Sep 2;11(7):540–51. doi: 10.1111/j.1567-1364.2011.00746.x (PMC3262967; doi:10.1111/j.1567-1364.2011.00746.x)
Supplement: Supplementary file 4 [file fyr0011-0540-SD4.docx]

Table S4. Tukey’s HSD (Honestly Significant Difference) p values for chemical attributes.

| **class 1** | **class 2** | **propanol** | **ethyl 2 methylbutyrate** | **isoamyl acetate** | **butanol** | **ethyl octanoate** |
| --- | --- | --- | --- | --- | --- | --- |
| paradoxus | wild | 0.858 | 0.121 | **0.015** | **0.009** | **0.001** |
| wine | wild | **0.024** | 0.273 | 0.572 | 0.910 | 0.996 |
| wine | paradoxus | 0.119 | **0.019** | **0.046** | **0.006** | **0.001** |
