# Supplementary material for: Divergence in wine characteristics produced by wild and domesticated strains of Saccharomyces cerevisiae
Source: FEMS Yeast Res. 2011 Sep 2;11(7):540–51. doi: 10.1111/j.1567-1364.2011.00746.x (PMC3262967; doi:10.1111/j.1567-1364.2011.00746.x)
Supplement: Supplementary file 5 [file fyr0011-0540-SD5.docx]

Table S5. Significant correlations between descriptive and chemical attributes.

| **comaprison type** | **attribute 1** | **attribute 2** | **correlation coefficient ^1^** | **P value** |
| --- | --- | --- | --- | --- |
| descriptive | cabbage | citrus | -0.69 | 0.029 |
| descriptive | cabbage | hay/straw | 0.67 | 0.035 |
| descriptive | cabbage | mushroom | 0.78 | 0.007 |
| descriptive | cabbage | oxidized | 0.81 | 0.005 |
| descriptive | cabbage | trueness | -0.65 | 0.044 |
| descriptive | citrus | mushroom | -0.76 | 0.011 |
| descriptive | citrus | trueness | 0.64 | 0.045 |
| descriptive | floral | trueness | 0.74 | 0.014 |
| descriptive | hay/straw | mushroom | 0.79 | 0.006 |
| descriptive | hay/straw | trueness | -0.62 | 0.056 |
| descriptive | mushroom | trueness | -0.78 | 0.007 |
| descriptive | oxidized | hay/straw | 0.66 | 0.039 |
| descriptive | oxidized | mushroom | 0.7 | 0.025 |
| descriptive | oxidized | trueness | -0.72 | 0.020 |
| descriptive | tree fruit | astringency | -0.67 | 0.036 |
| descriptive | wet dog | mushroom | 0.65 | 0.041 |
| chemical | A-amyl alcohol | acidity | 0.73 | 0.017 |
| chemical | acetaldehyde | phenyl.ethanol | 0.72 | 0.020 |
| chemical | butanol | ethyl octanoate | -0.86 | 0.004 |
| chemical | butanol | phenyl ethanol | 0.76 | 0.010 |
| chemical | butanol | volatile acidity (acetic) | -0.66 | 0.039 |
| chemical | ethyl acetate | isoamyl acetate | 0.7 | 0.025 |
| chemical | ethyl acetate | phenyl ethanol | -0.82 | 0.004 |
| chemical | ethyl acetate | volatile acidity (acetic) | 0.98 | 0.000 |
| chemical | ethyl hexanoate | ethyl octanoate | 0.74 | 0.014 |
| chemical | ethyl isobutyrate | isoamyl acetate | 0.69 | 0.027 |
| chemical | ethyl isobutyrate | isobutyl acetate | 0.68 | 0.030 |
| chemical | ethyl isobutyrate | total sulfur dioxide | -0.66 | 0.040 |
| chemical | ethyl propionate | isoamyl alcohol | -0.63 | 0.050 |
| chemical | ethyl propionate | isobutanol | -0.88 | 0.001 |
| chemical | ethyl-2-methylbutyrate | butanol | 0.82 | 0.004 |
| chemical | ethyl-2-methylbutyrate | dimethyl sulfide | -0.65 | 0.044 |
| chemical | ethyl-2-methylbutyrate | ethyl octanoate | -0.68 | 0.030 |
| chemical | ethyl-3-methylbutyrate | isoamyl alcohol | 0.65 | 0.042 |
| chemical | free sulfur dioxide | molecular sulfur dioxide | 0.96 | < 0.001 |
| chemical | free sulfur dioxide | total sulfur dioxide | 0.75 | 0.013 |
| chemical | free sulfur dioxide | trueness | 0.66 | 0.037 |
| chemical | isobutanol | dimethyl sulfide | 0.66 | 0.038 |
| chemical | isobutanol | isoamyl alcohol | 0.74 | 0.015 |
| chemical | isobutanol | pH | -0.65 | 0.044 |
| chemical | isobutyl acetate | dimethyl sulfide | 0.8 | 0.005 |
| chemical | isobutyl acetate | ethyl -2-methylbutyrate | -0.65 | 0.041 |
| chemical | molecular sulfur dioxide | total sulfur dioxide | 0.67 | 0.035 |
| chemical | molecular sulfur dioxide | trueness | 0.77 | 0.009 |
| chemical | pH | butterscotch | -0.87 | 0.001 |
| chemical | phenyl ethanol | volatile acidity (acetic) | -0.85 | 0.002 |
| chemical | propanol | pH | -0.64 | 0.049 |
| chemical | propanol | titratable acidity | 0.65 | 0.042 |
| chemical | total sulfur dioxide | titratable acidity | -0.7 | 0.024 |
| chemical | volatile acidity (acetic) | citrus | 0.64 | 0.046 |
| chemical and descriptive | acetaldehyde | citrus | -0.63 | 0.050 |
| chemical and descriptive | butanol | oxidized | 0.67 | 0.036 |
| chemical and descriptive | dimethyl sulfide | tree fruit | -0.67 | 0.033 |
| chemical and descriptive | ethyl acetate | astringency | 0.65 | 0.042 |
| chemical and descriptive | ethyl hexanoate | floral | 0.65 | 0.040 |
| chemical and descriptive | ethyl isobutyrate | tree fruit | -0.76 | 0.011 |
| chemical and descriptive | ethyl octanoate | floral | 0.73 | 0.017 |
| chemical and descriptive | ethyl-2-methylbutyrate | astringency | -0.74 | 0.015 |
| chemical and descriptive | ethyl-2-methylbutyrate | citrus | -0.69 | 0.027 |
| chemical and descriptive | isobutyl acetate | astringency | 0.7 | 0.023 |
| chemical and descriptive | isobutyl acetate | tree fruit | -0.86 | 0.001 |
| chemical and descriptive | propanol | acidity | 0.73 | 0.016 |
| chemical and descriptive | propanol | wet dog | -0.7 | 0.023 |
| chemical and descriptive | titratable acidity | acidity | 0.93 | < 0.001 |

^1^ correlation coefficient is Pearson’s r rank correlation coefficient
