# Supplementary material for: Divergence in wine characteristics produced by wild and domesticated strains of Saccharomyces cerevisiae
Source: FEMS Yeast Res. 2011 Sep 2;11(7):540–51. doi: 10.1111/j.1567-1364.2011.00746.x (PMC3262967; doi:10.1111/j.1567-1364.2011.00746.x)
Supplement: Supplementary file 6 [file fyr0011-0540-SD6.docx]

Table S6. The concentration of chemical compounds in wine produced by oak, palm, paradoxus, sake, wine and lab strains of *S. cerevisiae.*

|  |  | **yps1009** | **yps1000** | **yps163** | **yjm454** | **pw5** | **yps138** | **n17** | **k12** | **pr** | **m8** | **cdb** | **m33** | **w303** |
| --- | --- | --- | --- | --- | --- | --- | --- | --- | --- | --- | --- | --- | --- | --- |
|  |  | **oak** | **oak** | **oak** | **oak** | **palm** | **paradoxus** | **paradoxus** | **sake** | **wine** | **wine** | **wine** | **wine** | **lab** |
| basic chemistry^1^ | free sulfur dioxide (mg/L) | 4 | 5 | 4 | <2 | 7 | 4 | 6 | 5 | 4 | 8 | 5 | 3 | 10 |
|  | molecular sulfur dioxide (mg/L) | 0.21 | 0.25 | 0.2 | <0.10 | 0.42 | 0.23 | 0.29 | 0.26 | 0.25 | 0.45 | 0.28 | 0.22 | 0.52 |
|  | total sulfur dioxide mg/L | 89 | 92 | 77 | 84 | 81 | 98 | 101 | 81 | 78 | 109 | 96 | 77 | 100 |
|  | titratable acidity (g/L) | 4.6 | 4.5 | 4.6 | 4.6 | 5.4 | 4.6 | 4.2 | 4.4 | 5.1 | 4.5 | 4.2 | 5.2 | 4.2 |
|  | pH | 3.07 | 3.08 | 3.1 | 2.92 | 3.01 | 3.02 | 3.11 | 3.07 | 2.99 | 3.03 | 3.03 | 2.92 | 3.07 |
|  | volatile acidity (acetic) (g/L) | 0.32 | 0.22 | 0.24 | 0.09 | 0.33 | <0.05 | 0.14 | 0.09 | 0.34 | 0.13 | 0.3 | 0.21 | 0.16 |
| higher alcohols and fusel oils^1^ | acetaldehyde (mg/L) | 46 | 46 | 41 | 51 | 36 | 52 | 46 | 39 | 38 | 44 | 38 | 38 | 37 |
|  | ethyl acetate (mg/L) | 48 | 29 | 32 | 12 | 48 | <10 | 20 | 11 | 42 | 16 | 44 | 32 | 21 |
|  | methanol (mg/L) | 6 | 6 | 6 | <5 | 5 | 6 | <5 | <5 | 7 | 7 | <5 | <5 | 8 |
|  | 1-propanol (mg/L) | 23 | 21 | 20 | 31 | 29 | 24 | 25 | 34 | 32 | 28 | 30 | 48 | 32 |
|  | iso butanol (mg/L) | 47 | 34 | 46 | 149 | 56 | 29 | 41 | 19 | 58 | 79 | 26 | 68 | 48 |
|  | A-amyl alcohol (mg/L) | 16 | 16 | 22 | 18 | 25 | 20 | 17 | 15 | 20 | 20 | 13 | 20 | 18 |
|  | I-amyl alcohol (mg/L) | 106 | 102 | 147 | 156 | 131 | 117 | 91 | 62 | 104 | 143 | 70 | 134 | 87 |
| sulfides^1^ | hydrogen sulfide (ug/L) | <0.5 | <0.5 | <0.5 | <0.5 | <0.5 | <0.5 | <.5 | <0.5 | <0.5 | <0.5 | <0.5 | <0.5 | <0.5 |
|  | methyl mercaptan (ug/L) | <0.5 | <0.5 | <0.5 | <0.5 | <0.5 | <0.5 | <.5 | <0.5 | <0.5 | <0.5 | <0.5 | <0.5 | <0.5 |
|  | ethyl mercaptan (ug/L) | <0.5 | <0.5 | <0.5 | <0.5 | <0.5 | <0.5 | <.5 | <0.5 | <0.5 | <0.5 | <0.5 | <0.5 | <0.5 |
|  | dimethyl sulfide (ug/L) | 10.9 | 6.4 | 8 | 11.8 | 8.9 | 8 | 8.2 | 16.2 | 9.9 | 10.4 | 8.5 | 8 | 10.2 |
|  | dimethyl disulfide (ug/L) | <1.0 | <1.0 | <1.0 | 1.1 | 1.5 | <1.0 | <1.0 | 1.2 | <1.0 | <1.0 | <1.0 | <1.0 | <1.0 |
|  | diethyl sulfide (ug/L) | <0.5 | <0.5 | <0.5 | <0.5 | <0.5 | <0.5 | <0.5 | <0.5 | <0.5 | <0.5 | <0.5 | <0.5 | <0.5 |
|  | diethyl disulfide (ug/L) | <0.5 | <0.5 | <0.5 | <0.5 | <0.5 | <0.5 | <0.5 | <0.5 | <0.5 | <0.5 | <0.5 | <0.5 | <0.5 |
| alcohol percentage | alcohol % v/v | 8.55 | 8.71 | 8.60 | 8.69 | 8.52 | 8.71 | 8.83 | na | 10.48 | 8.80 | 8.38 | 8.57 | 9.12 |
|  | density (g/cm^3^) | 0.99577 | 0.99582 | 0.99562 | 0.99503 | 0.99604 | 0.99576 | 0.99531 | na | 0.99340 | 0.99397 | 0.99525 | 0.99565 | 0.99489 |
|  | alcohol % w/w | 6.77 | 6.90 | 6.82 | 6.89 | 6.75 | 6.91 | 7.00 | na | 8.33 | 6.99 | 6.64 | 6.79 | 7.23 |
| aroma compounds^2^ | acetaldehyde (mg/L) | 13.594 | 14.767 | 12.877 | 11.761 | 8.645 | 15.746 | 13.923 | na | 9.916 | 18.180 | 8.046 | 11.185 | 12.980 |
|  | ethyl acetate (mg/L) | 38.127 | 20.028 | 28.617 | 10.070 | 37.699 | 4.695 | 15.963 | na | 35.144 | 13.783 | 34.092 | 23.814 | 19.272 |
|  | Ethyl propionate (mg/L) | 0.223 | 0.212 | 0.228 | 0.158 | 0.224 | 0.248 | 0.205 | na | 0.237 | 0.203 | 0.234 | 0.224 | 0.198 |
|  | Ethyl isobutyrate (mg/L) | 0.153 | 0.145 | 0.153 | 0.172 | 0.179 | 0.088 | 0.134 | na | 0.155 | 0.131 | 0.088 | 0.182 | 0.105 |
|  | Isobutyl acetate (mg/L) | 0.154 | 0.000 | 0.088 | 0.161 | 0.157 | 0.000 | 0.000 | na | 0.154 | 0.095 | 0.000 | 0.088 | 0.000 |
|  | Ethyl butyrate (mg/L) | 3.251 | 3.503 | 3.151 | 3.088 | 2.750 | 2.505 | 2.462 | na | 4.087 | 2.866 | 2.345 | 2.719 | 4.041 |
|  | Propanol (mg/L) | 13.737 | 14.201 | 12.272 | 15.045 | 18.872 | 15.269 | 16.072 | na | 22.422 | 21.355 | 18.692 | 32.448 | 22.200 |
|  | Ethyl 2-methylbutyrate (mg/L) | 0.000 | 0.009 | 0.009 | 0.000 | 0.009 | 0.015 | 0.009 | na | 0.000 | 0.000 | 0.000 | 0.000 | 0.000 |
|  | Ethyl 3-methylbutyrate (mg/L) | 0.019 | 0.029 | 0.026 | 0.022 | 0.022 | 0.021 | 0.000 | na | 0.017 | 0.020 | 0.000 | 0.020 | 0.015 |
|  | Isobutanol (mg/L) | 32.115 | 33.300 | 30.611 | 124.528 | 41.817 | 19.746 | 29.200 | na | 40.740 | 59.732 | 17.076 | 49.613 | 36.305 |
|  | Isoamyl acetate (mg/L) | 0.045 | 0.033 | 0.053 | 0.041 | 0.064 | 0.000 | 0.023 | na | 0.043 | 0.027 | 0.034 | 0.040 | 0.025 |
|  | Butanol (mg/L) | 1.507 | 1.983 | 1.638 | 1.283 | 2.790 | 5.618 | 3.151 | na | 1.267 | 1.272 | 0.926 | 2.057 | 4.657 |
|  | Isoamyl alcohol (mg/L) | 80.906 | 105.039 | 106.080 | 140.305 | 113.166 | 98.176 | 76.608 | na | 72.655 | 134.332 | 57.591 | 115.382 | 75.893 |
|  | Ethyl hexanoate (mg/L) | 0.185 | 0.205 | 0.155 | 0.116 | 0.157 | 0.121 | 0.111 | na | 0.175 | 0.186 | 0.167 | 0.137 | 0.248 |
|  | Ethyl octanoate (mg/L) | 0.042 | 0.045 | 0.034 | 0.040 | 0.041 | 0.018 | 0.016 | na | 0.036 | 0.044 | 0.044 | 0.036 | 0.056 |
|  | Phenyl ethanol (mg/L) | 2.623 | 3.701 | 3.103 | 4.570 | 3.756 | 12.104 | 4.173 | na | 1.256 | 8.067 | 1.833 | 3.586 | 1.567 |

^1^ Chemical concentrations measured by ETS laboratory services (St. Helena, CA, USA).

^2^ Values reported are the mean of two replicate measurements.
